# Data mining of the transcriptome of *Plasmodium falciparum*: the pentose phosphate pathway and ancillary processes

**DOI:** 10.1186/1475-2875-4-17

**Published:** 2005-03-18

**Authors:** Zbynek Bozdech, Hagai Ginsburg

**Affiliations:** 1 School of Biological Sciences, Nanyang Technological University, 637551 Singapore; 2 Department of Biological Chemistry, Institute of Life Sciences, The Hebrew University of Jerusalem, Jerusalem 91904, Israel

## Abstract

The general paradigm that emerges from the analysis of the transcriptome of the malaria parasite *Plasmodium falciparum *is that the expression clusters of genes that code for enzymes engaged in the same cellular function is coordinated. Here the consistency of this perception is examined by analysing specific pathways that metabolically-linked. The pentose phosphate pathway (PPP) is a fundamental element of cell biochemistry since it is the major pathway for the recycling of NADP^+ ^to NADPH and for the production of ribose-5-phosphate that is needed for the synthesis of nucleotides. The function of PPP depends on the synthesis of NADP^+ ^and thiamine pyrophosphate, a co-enzyme of the PPP enzyme transketolase. In this essay, the transcription of gene coding for enzymes involved in the PPP, thiamine and NAD(P)^+ ^syntheses are analysed. The genes coding for two essential enzymes in these pathways, transaldolase and NAD^+ ^kinase could not be found in the genome of *P. falciparum*. It is found that the transcription of the genes of each pathway is not always coordinated and there is usually a gene whose transcription sets the latest time for the full deployment of the pathway's activity. The activity of PPP seems to involve only the oxidative arm of PPP that is geared for maximal NADP^+ ^reduction and ribose-5-phosphate production during the early stages of parasite development. The synthesis of thiamine diphosphate is predicted to occur much later than the expression of transketolase. Later in the parasite cycle, the non-oxidative arm of PPP that can use fructose-6-phosphate and glyceraldehyde-3-phosphate supplied by glycolysis, becomes fully deployed allowing to maximize the production of ribose-5-phosphate. These discrepancies require direct biochemical investigations to test the activities of the various enzymes in the developing parasite. Notably, several transcripts of PPP enzyme-coding genes display biphasic pattern of transcription unlike most transcripts that peak only once during the parasite cycle. The physiological meaning of this pattern requires further investigation.

## Introduction

The analysis of the transcriptome of *Plasmodium falciparum *has revealed that during the intraerythrocytic development of the parasite, genes coding for enzymes and proteins that are involved in complex cellular functions such as transcription, replication or energy metabolism, each involving many gene products, are transcribed in a coordinated fashion, supporting the notion that all components must be present at the right time to allow for optimal function [1-3]. While this may be true in general, it has already been found that scrutinizing the details of specific metabolic functions reveal some significant departures from this paradigm [4]. Such scrutiny has also provided some intriguing peculiarities that through detailed biochemical studies may reveal some parasite-specific functions that may enlighten our understanding of parasitism or even provide for novel targets for chemotherapeutic intervention. The present analysis explores of the transcriptome to fathom additional metabolic pathways.

**Figure 1:**
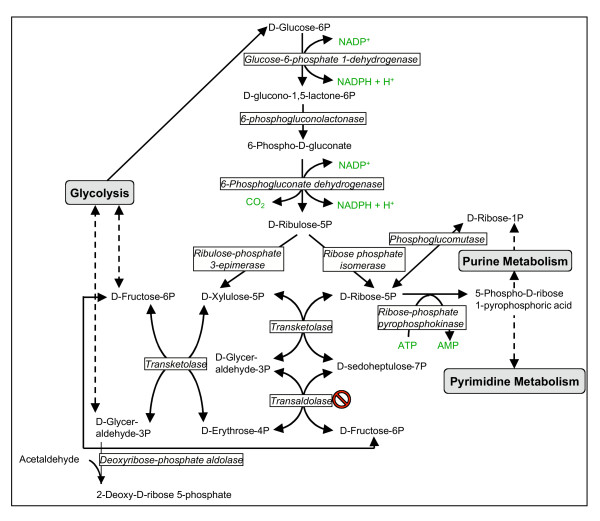
**Pentose phosphate pathway**. The no-entry symbol indicates that the gene coding for this enzyme could not be found in the genome of *P. falciparum*, but biochemical evidence suggests that the enzyme activity must be present. P represents phosphate.

During the erythrocytic stage the malaria parasite is engaged in intensive synthesis of nucleotides and is subjected to endogenously produced oxidative radicals that must be detoxified. Like other cells, in order to perform its anabolism, the parasite needs not only energy (ATP): it also needs reducing power, under the form of NADPH. Enzymes that function primarily in the reductive direction utilize NADP^+^/NADPH pair as co-factors as opposed to oxidative enzymes that utilize the NAD^+^/NADH cofactor pair. The conversion of ribonucleotides to deoxyribonucleotides (through the action of ribonucleotide reductase) requires NADPH as the electron source. Thus, any cell that proliferates rapidly requires large quantities of NADPH. NADPH can be produced during glucose-6-phosphate oxidation through the pentose-phosphate pathway (PPP; Figure 1). This pathway also produces ribose-5-phosphate (R5P), the sugar component of nucleic acids.

The reactions of the PPP operate exclusively in the cytoplasm. PPP has both an oxidative and a non-oxidative arm. The oxidation steps, utilizing glucose-6-phosphate (G6P) as the substrate, occur at the beginning of the pathway and are the reactions that generate NADPH. Thus, the first carbon of glucose-6-phosphate is first oxidized to a lactone (catalyzed by glucose-6-phosphate dehydrogenase) concomitantly releasing two electrons that reduce one molecule of NADP^+ ^to NADPH. The ensuing decarboxylation of 6-phospho-D-gluconate (catalyzed by 6-phosphogluconate dehydrogenase) releases two additional electrons, which reduce a second molecule of NADP^+^. A five-carbon sugar, D-ribulose-5-phosphate, is produced in the reaction. By isomerization, D-ribulose-5-phosphate is transformed into D-ribose-5-phosphate (R5P). To be used in nucleic acid synthesis, R5P is transformed into 5-Phospho-?-D-ribose 1-pyrophosphoric acid (PRPP) by ribose-phosphate diphosphokinase (EC: 2.7.6.1).

The non-oxidative reactions of the PPP are primarily designed to generate R5P. Equally important reactions of the PPP are to convert dietary 5 carbon sugars or D-ribose-1-phosphate generated in the salvage of purines (that can be slowly converted to R5P by phosphoglucomutase; EC: 5.4.2.2) into both 6 (fructose-6-phosphate) and 3 (glyceraldehyde-3-phosphate) carbon sugars which can then be utilized by the pathways of glycolysis. In the first reaction, R5P will accept two carbon atoms from xylulose-5-phosphate (obtained by epimerization of ribulose-5-P), yielding sedoheptulose-7-phosphate and glyceraldehyde-3-phosphate (catalyzed by transketolase). Sedoheptulose-7-phosphate transfers three carbons to glyceraldehyde-3-phosphate (catalyzed by transaldolase), yielding fructose-6-phosphate (F6P) and erythrose-4-phosphate. Erythrose-4-phosphate then accepts two carbon atoms from a second molecule of xylulose-5-phosphate (catalyzed again by transketolase), yielding a second molecule of F6P and a glyceraldehyde-3-P (GAP) molecule. F6P and a GAP can then enter glycolysis and eventually produce ATP. The intermediate erythrose-4-phosphate is a substrate for the shikimate pathway .

**Figure 2:**
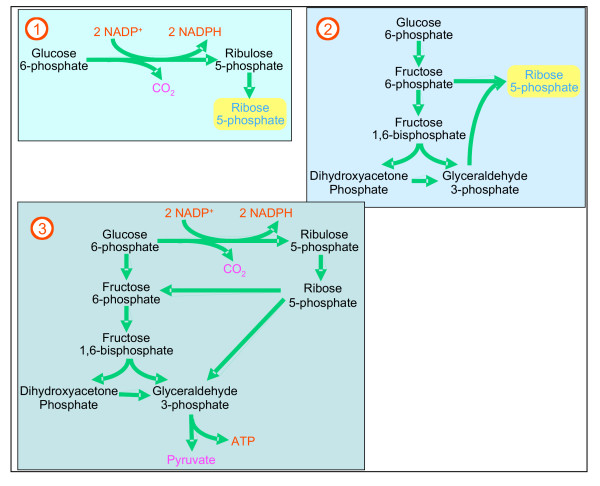
**Different modes of PPP action.** The PPP can function in different modes depending on the needs of the cell. Mode 1: Both ribose-5-phosphate and NADPH needed – predominating reactions are shown. – All the ribulose 5-phosphate is isomerized to ribose 5-phosphate, which is used for the synthesis of PRPP. Mode 2: More ribose-5-phosphate is needed than NADPH Ribose 5-phosphate is synthesized by the non-oxidative arm using fructose-6-phosphate and glyceraldehydes-3-phosphate supplied by glycolysis. Mode 3: The cell needs NADPH and ATP but *not *ribose-5-phosphate. Ribulose-5-phosphate is converted to fructose-6-phosphate and glyceraldehydes-3-phosphate which are channeled into glycolysis

The non-oxidative part of PPP can also work in the reverse direction utilizing fructose-6-phosphate glyceraldehyde-3-phosphate generated by glycolysis to produce ribose-5-phosphate. In essence, the PPP can operate in different modes (Figure 2). When both R5P and NADPH are needed (Figure 2 mode 1) only the oxidative path will operate: all the ribulose 5-phosphate is isomerized to R5P and the pathway is completed. When more ribose-5-phosphate than NADPH is needed (Figure 2 mode 2), R5P is synthesized by a reversion of the non-oxidative path from F6P and GAP generated by glycolysis. When the cell needs both NADPH and ATP but not R5P (Figure 2 mode 3): ribulose-5-phosphate is converted to F6P and GAP for glycolysis to make ATP and NADH.

The genes that code for enzymes participating in ancillary processes that produce NADP^+ ^and thiamine pyrophosphate, that serves as a co-factor for transketolase activity, should be expressed in coordination with the enzymes of the PPP. The details of the mentioned pathways can be also grasped at (; , respectively) and the time-dependent transcription of the genes coding for the different enzymes will be discussed below.

PPP activity in *P. falciparum-*infected erythrocytes has been measured [5-7]. The reverse activity of the non-oxidative arm of PPP has also been demonstrated by measuring the incorporation of radiolabel from [1-^14^C]glucose into nucleotides [6,8]. The former investigators have shown that 4/5 of the glucose incorporated into parasite nucleic acids comes from the condensation of F6P and GAP in the reverse action of this arm. Atamna *et al*, reported that infected cells have large increase of PPP activity where 82 % is contributed by the parasite while the host cell's PPP activity is activated some 24-fold as a result of the oxidative stress that the parasite generates and impinges on the host cell [9].

The gene coding for G6PD has been cloned [10] and the biochemical properties of the isolated enzyme have been characterized [11,12]. Molecular investigations have revealed that G6PD is coded by a hybrid gene that contains also the sequence of 6-phosphogluconolactonase (the second enzyme of PPP) [13,14]. Activity of 6-phosphogluconate dehydrogenase in *Plasmodium-*infected erythrocytes has been detected indirectly and alluded to a parasite enzyme, but not characterized [15-17]. Ribose-phosphate diphosphokinase of *P. falciparum *activity has been characterized and levels of its product 5'-phosphoribosyl-pyrophospate (PRPP) were measured in *P. falciparum-*infectederythrocytes [8,18]. The levels of PRPP were found to be increased 56-fold in infected cells at the trohozoite stage compared to uninfected erythrocytes.

The parasite contains substantial levels of the pyridine nucleotides NAD^+ ^and NADP^+ ^and their reduced forms [19] and jointly with the presence of genes coding for the enzymes necessary for their synthesis in the genome of *P. falciparum *(but see below the discussion concerning the absence of NAD^+ ^kinase-coding gene), indicate the ability of the parasite to produce these co-factors. NADP^+^/NADPH are used by several biochemical reactions in the parasite (Table 1), but the major role of NADPH is probably in the antioxidant defense of the parasite [20,21] (see  for details). Interestingly enough, the biochemical (such as the intermediate products, enzyme activities for each step, timing of production?) details of NAD(P)^+ ^synthesis in the parasite such as the intermediate products, enzyme activities for each step, timing of production, were not investigated.

**Table 1:** NADP utilizing enzymes Enzymes are arranged by their sequential functional order. They are given by their name, their EC numbers, the gene identification (PfID) in the *Plasmodium *genome database (PlasmoDB), the time (in hours post invasion (HPI)) in the parasite's developmental cycle when they are maximally transcribed obtained from the IDC database and the metabolic function of the enzyme.

Enzyme	EC number	PfID	Peak (HPI)	Metabolic function
3-oxoacy1-[acy1-carrier-protein] reductase	1.1.1.100	PFI1125c	28	Fatty acid synthesis
1-deoxy-D-xylulose-5-phosphate reductoisomerase	1.1.1.267	PF14_0641	30	Isoprenoid metabolism
Isocitrate dehydrogenase (NADP+);	1.1.1.42	PF13_0242	28	TCA cycle
Phosphogluconate dehydrogenase	1.1.1.44	PF14_0520	26	PPP
Glucose-6-phosphate 1-dehydrogenase;	1.1.1.49	PF14_0511	48	PPP
Pyruvate dehydrogenase (acetyltransferring).	1.2.4.1	PF11 0256 PF14 0441	28 28	Pyruvate metabolism
Glutamate dehydrogenase (NADP)	1.4.1.4	PF14 0164 PF14 0286	1 30	Glutamate metabolism
Pyrroline-5-carboxylate reductase	1.5.1.2	MAL13P1.284	18	Proline metabolism
Dihydrofolate reductase	1.5.1.3	PFD0830w	26	Folate synthesis
NAD(P)+ transhydrogenase (B-specific);	1.6.1.1	PF14_0508	32	?
NADPH hemoprotein reductase	1.6.2.4	PFI1140w	13	Flavoprotein reduction
Dihydrolipoamide dehydrogenase	1.8.1.4	PF08 0066 PFL1550w	28 11	Pyruvate metabolism
Dihydrolipoamide S-acetyltransferase;	2.3.1.12	PF10 0407	28	Pyruvate metabolism

In this *in silico *analysis, the stage-dependent transcription of genes that code for enzymes that are involved in the PPP activity of the parasite will be analyzed in a functional context.

## Materials and methods

The expression data used in this study was obtained from the transcriptome database  of the *P. falciparum *intraerythrocytic developmental cycle as described [2]. This database contains the relative mRNA abundance for every hour of the intraerythrocytic cycle of parasite development based on the 70-mer oligonucleotide microarray [22]. The expression profile of each transcript is represented by an array of ratios between the mRNA level in the time point sample versus a fixed mRNA level in the control RNA pool. Loess smoothed expression profiles used in this study were calculated as described (see [2]). The peak hour induction was calculated from these profiles. This represents more accurate estimate of the timing of maximal expression since the loess smoothing provides and average mRNA abundance values over a time interval, correcting possible minor fluctuations in the raw value profiles. Each expression profile was subsequently normalized by its peak value. It is assumed that for each metabolic pathway there is a stoichiometric relationship between its individual enzymes and therefore the normalized values are more meaningful for functional evaluation. All enzymes discussed in this essay with their EC numbers and gene ID's are shown in Table 2. Amplification of transcription was calculated by dividing the maximum value by the minimum value of the profile of relative mRNA abundance. A fair agreement between the time-dependent transcription data of the DeRisi transcriptome and Winzeler's data has been observed (data not shown).

**Table 2:** Enzymes of the pentose phosphate pathway and pyridine nucleotide metabolism. Enzymes are grouped into pathways. They are given by their name, their EC numbers, the gene identification (PfID) in the *Plasmodium *genome database (PlasmoDB)), the time (in hours post invasion (HPI)) in the parasite's developmental cycle when they are maximally transcribed obtained from the IDC database and the metabolic function of the enzyme.

Pathway	Enzyme	EC number	Pf ID	Peak (HPI)
Pentose phosphate pathway	Glucose-6-phosphate dehydrogenase	1.1.1.49	PF14_0511	48
	6-phosphogluconolactonase	3.1.1.31	PF14 0511	48
	6-phosphogluconate dehydrogenase	1.1.1.44	PF14_0520	26
	Ribulose 5-phosphate 3-epimerase	5.1.3.1	PFL0960w	29
	Ribose 5-phosphate isomerase	5.3.1.6	PFE0730c	21
	Transketolase	2.2.1.1	MAL6P1.110	17
	Ribose phosphate diphosphokinase	2.7.6.1	PF13 0143 PF13 0157	21 21
	Deoxyribose phosphate aldolase	4.1.2.4	PF10 0210	21
	Phosphoglucomutase	5.4.2.2	PF10 0122	21

Pyridine nucleotide metabolism	Nicotinamidase	3.5.1.19	chr3.glm 243	18, 38
	Nicotinatephosphoribosyl transferase	2.4.2.11	chr6.glm_337	25
	Nicotinate-nucleotide adenylyl transferase	2.7.7.18	chr13.glm_329	20, 40
	NAD^+ ^synthetase	6.3.5.1	PFI1310w	28
	Pyridine nucleotide transhydrogenase	1.6.1.1.	PF14_0508	32

Thiamine metabolism	Hydroxyethylthiazole kinase	2.7.1.50	PFL1920c	22
	Hydroxymethylpyrimidine kinase/ Phosphomethylpyrimidine kinase	2.7.149 2.7.4.7	PFE1030c	33
	Thiamine-phosphate diphosphorylase	2.5.1.3	MAL6P1.285	1
	Thiamine diphosphokinase	2.7.6.2	PFI1195c	28

## Results and Discussion

At the onset of the present analysis, it should be underscored that time-dependent transcription does not always overlaps translation. Hence, transcript levels cannot be directly extrapolated to levels of their translated product. Moreover, transient transcription does not divulge on the stability of the translated products. Indeed, a recent analysis has shown for several genes that while the transcript peaks at the trophozoite stage and declines thereafter, the translated protein continues to accumulate [23]. Significant discrepancies between mRNA and protein abundance in *P. falciparum *were also reported by LeRoche *et al*. [24]. This was shown to be due to a delay between the maximum detection of an mRNA transcript and that of its cognate protein. Surely, a protein cannot be produced if its cognate gene is not transcribed. Therefore, the present analysis can at best set a time for possible translation but not for its actual occurrence. Thus, while transcript levels are informative for the concerted action of metabolically related enzymes, post-transcriptional mechanisms for controlling protein levels and protein stability must also be considered.

In this analysis, the transcription of genes coding for enzymes that constitute the PPP will be discussed first followed by those that are involved in the synthesis of NAD(P)^+ ^and of thiamine pyrophosphate.

### The pentose phosphate pathway

**Figure 3:**
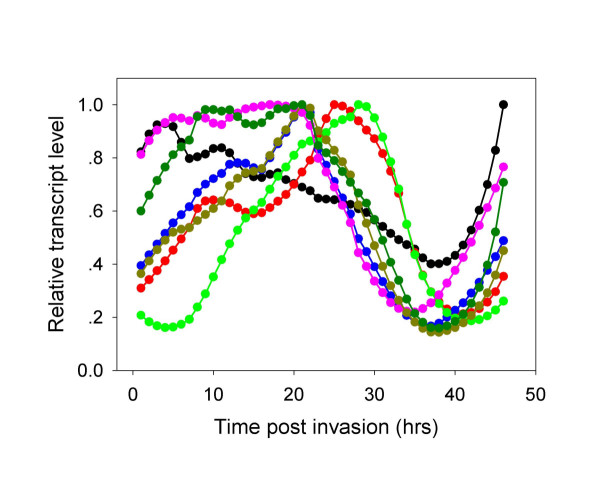
**Time dependent transcription of genes coding for enzymes involved in the pentose phosphate pathway**. Glucose-6-phosphate dehydrogenase/6-phosphogluconolactoase – black filled circles; 6-phosphogluconate dehydrogenase – red filled circles; Ribose phosphate isomerase – blue filled circles; Ribulose 5-phosphate epimerase – green filled circles. Transketolase – purple filled circles; Ribose phosphate diphosphokinase (PF13_0157) – gold filled circles and (PF13_0143) – dark green filled circles. Glucose-6-phosphate dehydrogenase and 6-phosphogluconolactoase were grouped together because they constitute a chimerical protein.

The pathway is shown in Figure 1 and the time-dependent transcription of the different genes is depicted in Figure 3. Whereas G6PD (EC: 1.1.1.49) and hence its conjoint 6-phosphogluconolactonase (EC: 3.1.1.31) are transcribed at high levels during the early stages of parasite development, the transcription of 6-phosphogluconate dehydrogenase (EC: 1.1.1.44) seems to be biphasic (first peak at 9 hours post invasion (HPI) while the major peak is observed at 26 HPI). The two other enzymes that need to be present for full synthesis of PRPP are ribose phosphate isomerase (EC: 5.3.1.6) and ribose-phosphate diphosphokinase (EC: 2.7.6.1). The first has a small peak at 12 HPI and a major one at 21 HPI. The parasite contains 2 genes that code for ribose-phosphate diphosphokinase (PF13_0143 and PF13_0157). Both peak at 21 HPI. Thus, the oxidative arm seems to be fully activated to meet the requirement of ribonucleotide synthesis according to mode 1 of Figure 2. The genes that code for this process are coordinately transcribed starting immediately after invasion and peak at 12 HPI [2]. Biochemical evidence indicates that ribonucleotide synthesis starts at the mature ring stage [25]. Functioning according to mode 1 (Figure 2) would also supply reducing power to neutralize the toxic reactive oxidative species that are maximally produced during the hemoglobin-rich cytosol of the host cell [9]. It seems that the synthesis of PRPP and its utilization in the synthesis purine and pyrimidine nucleotides serves as a metabolic sink that could accelerate the pace of the oxidative arm. Contrary to this judicious coordination, the transcription of genes coding for enzymes needed for the synthesis of NADP^+ ^that is essential for the operation of the oxidative arm, lags considerably behind (see below). This is a crucial paradox that waits to be resolved.

The situation of the non-oxidative arm genes is different. The gene that codes for ribose phosphate isomerase (EC: 5.1.3.1) peaks at 21 HPI and this seems to restrict the expression of the non-oxidative arm, since transketolase (EC: 2.2.1.1) is transcribed maximally immediately following invasion. Most importantly, the gene that codes for transaldolase (EC: 2.2.1.2) could not be found in the genome of *P. falciparum *(or in any other *Plasmodium *species sequenced so far, or in any other Apicomplexans for that matter) thus precluding the completion of the analysis of the non-oxidative arm transcription. As mentioned above, biochemical evidence indicates that this arm is active in the parasite. If it is indeed fully activated when ribose phosphate isomerase is transcribed (and supported by the transcription of the genes that code for ribose-phosphate diphosphokinase that peak at 21 HPI), it would match the transcription of the deoxyribonucleotide synthesis-related genes that starts at 18 HPI and peaks at 30 HPI [2] as well as those related to NADP^+ ^synthesis. When the synthesis of ribonucleotides and deoxyribonucleotides ebbs off towards the end of the intraerythrocytic life cycle of the parasite, the PPP probably functions according to mode 3 (Figure 2) providing both NADPH and ATP. A feature that emerges from this analysis is that the transcription of the genes that code for enzymes acting in the non-oxidative arm are coordinated as shown for other clusters of genes whose products are functionally related [1-3]. However, it remains to be seen if the cognate enzymes of these transcripts are similarly coordinated since significant discrepancies between mRNA and protein abundance has been recently reported [24].

### NAD(P) biosynthesis

**Figure 4:**
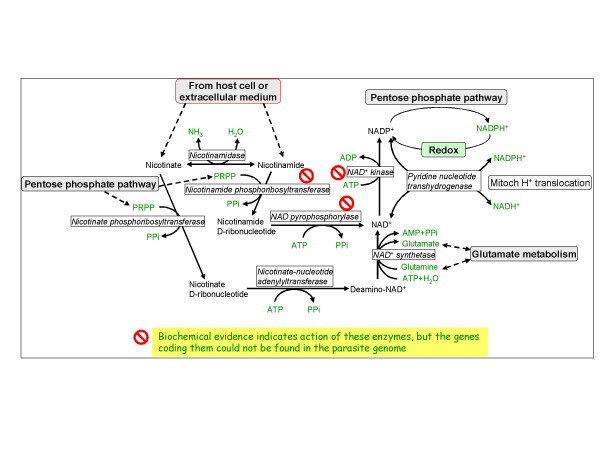
**Nicotine nucleotide metabolism**. The no-entry symbol indicates that the gene coding for this enzyme could not be found in the genome of *P. falciparum*, but biochemical evidence suggests that the enzyme activity must be present.

**Figure 5:**
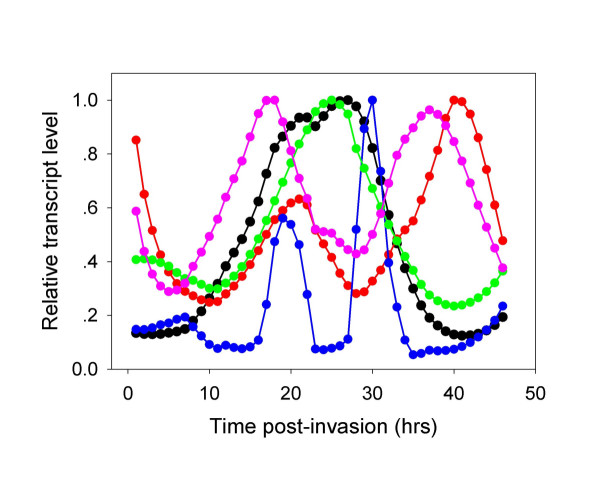
**Time dependent transcription of genes coding for enzymes involved in the biosynthesis of NAD(P)^+^**. Nicotinamidase – purple filled circles; Nicotinate phosphoribosyl transferase – green filled circles; Nicotinate-nucleotide adenylyltransferase – red filled circles; NAD^+ ^synthetase – black filled circles; NAD(P) transhydrogenase – blue filled circles.

The pathway is shown in Figure 4 and the time-dependent transcription of the different genes is depicted in Figure 5. Nicotine adenine dinucleotide (NAD^+^) and NAD phosphate (NADP^+^) play key roles in glycolysis and PPP as well as many other enzymatic reactions (see Tables 3 and 1, respectively). NAD synthesis starts by converting nicotinic acid to nicotinate D-ribonucleotide by nicotinate phosphoribosyltransferase (NAPRT; EC: 2.4.2.11). Nicotinate D-ribonucleotide is then adenylated to deamino-NAD+ in a reaction catalyzed by nicotinate-nucleotide adenylyltransferase (EC: 2.7.7.18) then this intermediate is amidated to NAD^+ ^by NAD synthetase (EC: 6.3.5.1). Nicotinamide can be used for NAD synthesis either by being deamidated to nicotininc acid by nicotinamidase (EC: 3.5.1.19) or by being converted to nicotinamide mononucleotide by nicotinamide phosphoribosyltransferase (NPRT; EC: 2.4.2.12) and then to NAD by NAD pyrophosphorylase (EC: 2.7.7.1). Genes coding for the two latter enzymes could not be found in the genome *of P. falciparum*, although a measurable activity of NPRT has been detected [26]. These enzymes are present in normal erythrocytes and at physiological conditions the production of NAD^+ ^from nicotinamide seems to be more important than that of NADP^+ ^[27]. Zerez *et al*. [26] demonstrated 15-fold increase in the levels of NAD^+ ^(NAD^+ ^+ NADH) in *P. falciparum*-infected erythrocytes, as well as 3-fold increase in NAPRT activity. These results indicate that the parasite is capable of NAD synthesis although neither the activity of nicotinate-nucleotide adenylyltransferase or of NAD synthetase were increased in infected cells compared to uninfected erythrocytes. This observation suggests that these enzymes must be active in the parasite since the digestion of host cell cytosol during parasite development should have reduced their activities. Abundant nicotinamidase activity was also detected in infected cells implying that the parasite can synthesize NAD^+ ^from both nicotinic acid and nicotinamide (both present in RPMI-1640 medium used for parasite cultivation). The concentrations of NADP^+ ^in infected cells are 10-fold lower that those of NAD^+^. A gene coding for NAD^+ ^kinase could not be found in the genome of *P. falciparum *or in any other *Plasmodium *species or in other Apicomplexans, but could be detected in *Giardia lamblia*. No information about the action of NAD kinase in the parasite could be found.

**Table 3:** NAD^+^-utilizing enzymes. Enzymes are arranged by their sequential functional order. They are given by their name, their EC numbers, the gene identification (PfID) in the *Plasmodium *genome database (PlasmoDB), the time (in hours post invasion (HPI)) in the parasite's developmental cycle when they are maximally transcribed obtained from the IDC database, the metabolic function of the enzyme.

Enzyme	EC number	PfID	Peak (HPI)	Metabolic function
Pyrroline carboxylate reductase	1.5.1.2	MAL13P1.284	18	Methionine polyamine metabolism
Ferrodoxin reductase-like protein	1.7.1.4	PF07_0085	21	Nitrogen metabolism
Glutamate dehydrogenase	1.4.1.2	PF08_0132	20	Glutamine metabolism
Aminomethyltransferase	2.1.2.10	PF13 0345	22	Folate biosynthesis
L-lactate dehydrogenase	1.1.1.27	PF13 0141	22	Glycolysis
Enoyl-acyl carrier reductase	1.3.1.9	MAL6P 1.275	30	Fatty acid synthesis
2-oxoglutarate dehydrogenase el component	1.2.4.2	PF08_0045	30	TCA cycle
Lipoamide dehydrogenase, putative	1.8.1.4	PF08_0066	28	TCA cycle
Pyruvate dehydrogenase El component, ?-subunit	1.2.4.1	PF11_0256	28	Fatty acid synthesis
NADH-cytochrome b5 reductase	1.6.2.2	PF13_0353	32	Electron transport
Malate dehydrogenase, putative	1.1.1.37	MAL6P 1.242	34	Pyruvate metabolism
Glycerol-3-phosphate dehydrogenase	1.1.1.8	PF11 0157 PFL0780w	37 19	Glycolysis; Glycerol metabolism
GDP-mannose 4,6-dehydratase	1.1.1.187	PF08_0077	39	Mannose and fructose metabolism
Inosine-5 '-monophosphate dehydrogenase	1.1.1.205	PFI1020c	12	Purine metabolism
3-methyl-2-oxobutanoate dehydrogenase (lipoamide)	1.2.4.4	PFE0225w	32	Leucine, isoleucine and valine degradation
Nitrate reductase	1.7.1.1	chr13.glm_739	32	Nitrogen metabolism

Inspection of the time-dependent transcription of genes coding enzymes that are involved in NAD(P)^+ ^biosynthesis reveal an unusual pattern. Transcriptional profiles of at least three transcripts show two peaks, namely those of nicotinamidase, of nicotinate-nucleotide adenylyltransferase and of NAD(P)^+ ^transhydrogenase, as compared to most other transcripts in the parasite transcriptome that are monophasic. The reason for this pattern is unclear as biochemical data are uanavilable for any physiological intepretation, but it may suggest that the the enzymes are not stable. Assuming that the first smaller peak of nicotinate-nucleotide adenylyltransferase (20 HPI) is sufficient for adequate expression of enzymatic activity, the biosynthesis of NAD(P)^+ ^should peak when NAD^+ ^synthetase is at its peak, i.e., at 28 HPI. Thus, by 28 HPI the transcripts of all enzymes necessary for NAD^+ ^synthesis are fully deployed and somewhat later, synthesis itself is probably fully deployed. This time pattern means that important activities, such as glycolysis (the transcription of genes coding for glycolytic enzymes starts to peak at 9 HPI and starts to decrease at 24 HPI [2] At earlier stages glycolysis and PPP activity probably depend on the pool of NAD(P)^+ ^that were present in the invading merozoite. For the full activation of glycolysis at the trophozoite stage [28], the pool of NAD(P)^+ ^has to be significantly amplified. As can be seen in Table 3, several genes coding for enzymes requiring NAD(H) are transcribed earlier than the complete transcription of genes related to NAD^+ ^synthesis. Such an outstanding exception is that of inosine-5'-monophosphate dehydrogenase, an essential enzyme in the purine metabolic pathway: it is transcribed too early to allow its translated product to be functionally useful. The functional meaning of the second peaks of nicotinamidase and of nicotinate-nucleotide adenylyltransferase is enigmatic since the transcription of all other enzyme-coding genes declines to a minimum when they peak. For being meaningful physiologically, the stability of all other enzymes must be sustained with time. Indeed, it has been recently shown that the levels of some proteins such as methionine adenosyltransferase, ornithine aminotransferase, lactate dehydrogenase, glyceraldehyde 3-phosphate dehydrogenase and enolase, are not only increased following transcription, but further increase after their transcript levels decline [23]. Finally, NAD^+ ^kinase is essential for the production of NADP^+^. Its absence from the genome is indeed very perplexing because biochemical evidence indicate that the parasite is able to synthesize NADP^+ ^[19] and the presence of many enzymes that need it as a cofactor, implicate that its synthesis is essential for parasite growth. As long as the gene (or some surrogate mecahnism) is not identified, nothing can be said about the coordination of NADP^+ ^biosynthesis and the expression of enzymes that utilize it (Table 1).

Transhydrogenase operates at an important interface between NAD(H) and NADP(H) and between the mitochondrial proton electrochemical gradient ??; [29]. Under regular physiological conditions, the enzyme is a consumer of ??:

NADH + NADP^+ ^+ H^+^_out_ _NAD^+ ^+ NADPH + H^+^_in_.

The energy of the gradient can drive the [NADPH][NAD^+^]/[NADP^+^][NADH] ratio to values >400. Transhydrogenation can also function in the reverse direction from NADPH to NAD^+^. This is accompanied by outward proton translocation and formation of ??. In this mode, the enzyme utilizes substrate binding energy for proton pumping. Therefore, in terms of energy transduction, the transhydrogenase works in principle like the ATP synthase complex of mitochondria, the proton ATPase. Given the fact that the parasite genome does not have the full complement of genes coding for the mitochondrial proton ATPase [30], it is tempting to suggest that the transhydrogenase could fulfill such role. The transcription of the gene coding for transhydrogenase shows two very distinct peaks at 20 and 30 HPI, with no detectable transcription between them. It may well be that the second peak is adjusted to the time of elongation and division of the single mitochondrion [31].

### Thiamine biosynthesis

**Figure 6:**
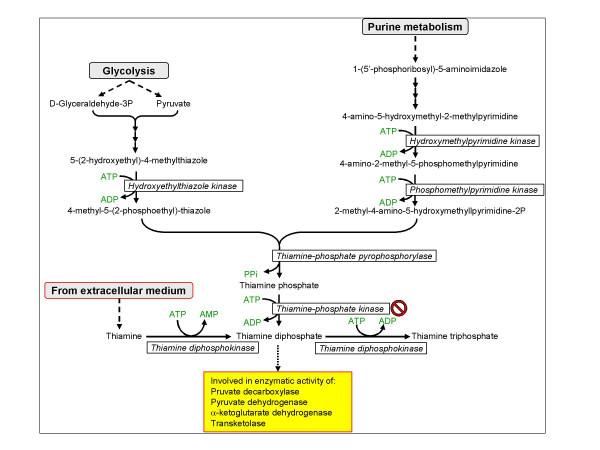
**Biosynthesis of thiamine diphosphate**. The no entry symbol indicates that the gene coding for this enzyme could not be found in the genome of *P. falciparum*. No biochemical evidence exists to confirm or refute enzyme activity. It has been added to the scheme because genes coding for enzymes preceding it in the pathway are present in the genome.

**Figure 7:**
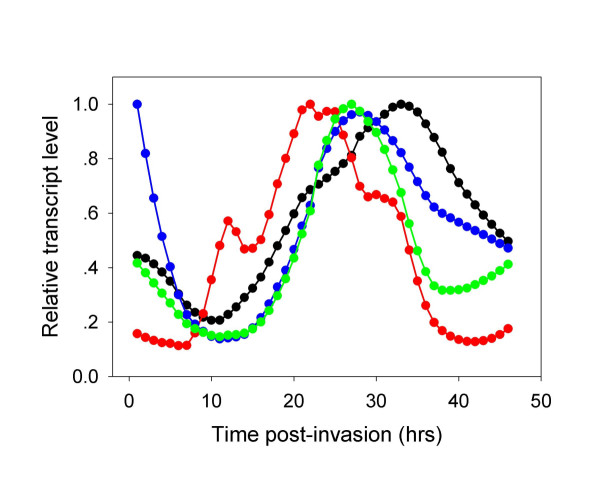
**Time dependent transcription of genes coding for enzymes involved in the biosynthesis of thiamine diphosphate**. Hydroxyethylthiazole kinase – red filled circles; Hydroxymethylpyrimidine kinase/phosphomethylpyrimidine kinase – black filled circles; Thiamine-phosphate diphosphorylase – blue filled circles; Thiamine diphosphokinase – green filled circles. Hydroxymethylpyrimidine kinase and phosphomethylpyrimidine kinase were grouped together because they probably constitute a chimerical protein.

The essential product of this pathway, thiamine diphosphate, is a cofactor of many enzymes (Table 4). The pathway is shown in Figure 6 and the time-dependent transcription of the different genes is depicted in Figure 7. Thiamine (vitamin B_1_) is the precursor of the coenzyme thiamine pyrophosphate that is involved in the action of many decarboxylating enzymes and relevant to the present analysis, in the function of transketolase. *P. berghei*-infected erythrocytes contain higher levels of thiamine than their uninfected counterparts [32] and thiamine deficiency retards the propagation of this parasite *in vivo *[33]. The incidence of thiamine deficiency in adults admitted to hospital with malaria in Thailand has been examined [34] and it was observed that in hospitalized thiamine deficiency commonly complicates acute falciparum malaria, particularly in severe infections, and could contribute to dysfunction of the central nervous system. Thiamine could be obtained by the parasite from the extracellular space (like in animal cells) and pyrophosphorylated by thiamine diphosphokinase (EC: 2.7.6.2). However the presence of genes coding for enzymes that participate in the eukaryote biosynthetic pathway of thiamine phosphate, suggest that the parasite is able to synthesize its own precursor. Nevertheless, the absence of a gene coding for thiamine phosphate kinase (EC: 2.7.4.16) must be resolved and direct biochemical demonstration of activity must be performed before a firm statement can be made on this metabolic pathway. It is not unlikely that thiamine phosphate kinase is not needed, as is the situation in yeast [35]. There it is suggested that thiamine phosphate is hydrolyzed to thiamine by a non-specific phosphatase and thiamine is then converted to thiamine pyrophosphate by thiamine diphosphokinase.

**Table 4:** Thiamine diphosphate utilizing enzymes. Enzymes are arranged by their sequential functional order. They are given by their name, their EC numbers, the gene identification (PfID) in the *Plasmodium *genome database (PlasmoDB), the time (in hours post invasion (HPI)) in the parasite's developmental cycle when they are maximally transcribed obtained from the IDC database, the metabolic function of the enzyme.

Enzyme	EC number	PfID	HPI	Metabolic function
Pyruvate dehydrogenase E1	1.2.4.1	PF11_0256 (a) PF14_0441 (b)	28 28	Apicoplast fatty acid synthesis
Transketolase	2.2.1.1	MAL6P1.110	17	Pentose phosphate pathway
Oxalyl-CoA decarboxylase	4.1.18	MAL6P1.231 PFF0945c	26 NA	Glyoxylate and dicarboxylate metabolism
1-deoxy-D-xylulose-5-phosphate synthase	2.2.1.7	PF13_0207	21	Isoprenoid biosynthesis
3-methyl-2-oxobutanoate dehydrogenase	1.2.4.4	PFE0225w	32	Leucine, isoleucine and valine degradation
Oxoglutarate dehydrogenase	1.2.4.2	PF08_0045	32	Mitochondrial TCA cycle

The transcription of all genes coding for enzymes involved in thiamine pyrophosphate seems to be coordinated, single phased and peaking between 20 and 30 HPI. If thiamine is obtained from the host and thiamine pyrophosphate is synthesized by the single step mediated by thiamine diphosphokinase, the peak transcription of this gene at 28 HPI lags by several hours after that of transketolase (17 HPI), but since transketoalse is not the expression time-setter of PPP, this lag does not seem to limit the full activity of PPP. However, if most of the thiamine is generated endogenously by the parasite, the supply of this precursor will peak only at 33 HPI, thus limiting full PPP activity. It is not unlikely that both processes occur in tandem or that at advanced stage of parasite development, the need for thiamine cannot be met anymore by exogenous supply and the endogenous synthesis joins the game.

## Conclusion

The analysis of time-dependence transcription of parasite genes concluded that the parasite has evolved a highly specialized mode of transcriptional regulation that produces a continuous cascade of gene expression, beginning with genes corresponding to general cellular processes, such as protein synthesis, and ending with *Plasmodium*-specific functionalities, such as genes involved in erythrocyte invasion [2]. However, a meticulous analysis shows marked and important deviations from this prototype that reveal a lack of coordinated transcription of genes coding for enzymes of the same metabolic pathway and between pathways. There are three most straightforward explanations for these apparent discrepancies. First, there are additional enzymes facilitating the "missing" activities and their identity was not revealed by the present annotations due to their diverse amino acid sequence. Second, the misaligned transcriptional regulation reflect an intricate interplay of the biosynthetic pathways where delayed production of metabolites in one pathway functions as a rate limiting factor for other pathway, which is otherwise fully deployed. Last but not least, post-transcriptional regulation may also play a role. All theories create an intriguing possibility for further studies. Clearly this effort will be enhanced by substantial progress in proteomics and most importantly, direct biochemical demonstrations of activities of individual enzymes and entire pathways.

## Authors' contributions

Each author contributed equally to this investigation.
